# Bovine Milk Protein-Derived Preparations and Their Hydrolysates as Sources of ACE-Inhibitory, DPP-IV-Inhibitory, and Antioxidative Peptides Analyzed Using in Silico and in Vitro Protocols

**DOI:** 10.3390/ijms26094323

**Published:** 2025-05-01

**Authors:** Anna Iwaniak, Piotr Minkiewicz, Damir Mogut, Justyna Borawska-Dziadkiewicz, Justyna Żulewska, Małgorzata Darewicz

**Affiliations:** 1 Department of Food Biochemistry, Faculty of Food Science, University of Warmia and Mazury in Olsztyn, Pl. Cieszyński 1, 10-719 Olsztyn-Kortowo, Poland; minkiew@uwm.edu.pl (P.M.); damir.mogut@uwm.edu.pl (D.M.); justyna.borawska@uwm.edu.pl (J.B.-D.); darewicz@uwm.edu.pl (M.D.); 2 Department of Dairy Science and Quality Management, Faculty of Food Science, University of Warmia and Mazury in Olsztyn, Oczapowskiego 7, 10-719 Olsztyn-Kortowo, Poland; justyna.zulewska@uwm.edu.pl

**Keywords:** bioactive peptides, BIOPEP-UWM database, hydrolysates, milk proteins, milk preparations

## Abstract

Bovine milk protein preparations (MPPs), namely micellar casein concentrate (MCC), serum protein concentrate (SPC), and MCC with ultrafiltrated buttermilk permeate (MBP), were analyzed as sources of inhibitors of angiotensin-converting enzyme (i.e., ACE) and dipeptidylpeptidase IV (i.e., DPP-IV) as well as antioxidative peptides. The studies involved in silico predictions of the release of biopeptides from bovine milk proteins. Then, all MPPs were subjected to the simulated gastrointestinal digestion using the INFOGEST protocol. Results using a BIOPEP-UWM database tool indicated that 59 biopeptides exhibiting the above-mentioned activities could be produced upon the action of pepsin, trypsin, and chymotrypsin. Thirty-six biopeptides were identified in at least one of the three MPPs subjected to the INFOGEST protocol. MCC before simulated digestion exhibited the strongest ACE-inhibiting activity among all MPPs (IC_50_ = 1.856 mg/mL). The weakest ACE inhibitory effect was demonstrated for MBP after duodenal digestion (i.e., MBP D; IC_50_ = 7.627 mg/mL). The above MPP showed the strongest DPP-IV-inhibiting activity (IC_50_ = 0.0067 mg/mL). All MPPs exhibited antioxidative activity, with the strongest ABTS^•+^ (i.e., 2,2′-azino-bis(3-ethylbenzotialozline-6-sulfonic acid) radical scavenging effect shown for MBP D (IC_50_ = 2.754 mg/mL), and the strongest DPPH^•^ (i.e., 2,2-diphenyl-*β*-picrylhydrazyl) radical scavenging activity (IC_50_ = 1.238 mg/mL) demonstrated for SPC D. Among all MPPs, SPC D also exhibited the highest FRAP (i.e., Ferric Reducing Antioxidant Power) bioactivity (IC_50_ = 13.720 mg/mL), whereas MBP D was the MPP with the lowest FRAP potential (IC_50_ = 20.140 mg/mL). The present study results show the potential of all MPPs as functional additives to support health-beneficial functions of dairy products.

## 1. Introduction

Mother Nature provides a human being (and other beings) with the most complete food—milk [1], which is produced by various species like humans, dairy cows, buffalo, goats, sheep, camels, yaks, etc. [2]. Among them, 85% of the world’s milk production comes from cattle [3]. The origin of milk may affect its chemical composition; however, it possesses health-beneficial and functional properties, regardless of species [2]. The health promoting value of milk is owed to its biologically-active nutrients like, e.g., high quality and easily absorbable protein [4]. Moreover, due to their properties, like solubility, water binding, viscosity, and heat stabilization, proteins affect the functionality of milk and dairy products [2].

According to McBean et al. [5], the nutritional benefits of bovine milk proteins result from the presence of nine essential amino acids, necessary for human growth and tissue functions. Moreover, the health-supportive value of bovine milk proteins is associated with exogenous peptides produced upon the action of proteolytic enzymes [6]. According to Marcone et al. [7], such peptides were proven to, e.g., reduce blood pressure, regulate glucose level and lipid concentration, as well as exert antioxidative and other effects [7]. The activities of peptides involved in the regulation of blood pressure, sugar, and lipid levels are related to the inhibition of enzymes naturally occurring in the human body [8]. Briefly, peptidic inhibition of the angiotensin-converting enzyme (ACE, EC 3.4.15.1) leads to blood pressure decrease by hindering vasoconstriction of angiotensin II and vasodilation of bradykinin [9]. In turn, peptides inhibiting dipeptidyl peptidase IV (DPP-IV; EC 3.4.14.5) are responsible for blocking the degradation of two endogenous peptides by this enzyme: glucagon-like peptide 1 (GLP-1) and gastric inhibitory peptide (GIP), leading to the prolongation of their half-lives and their increased plasma concentration, and the reduction in the postprandial blood glucose level [10]. Therefore, peptides with ACE-inhibitory and DPP-IV-inhibitory activity are deemed valuable diet components, acting as hypotensive, hypoglycemic, and anti-obesity agents, respectively [11,12]. According to Sarmadi and Ismail [13], metabolic syndrome dysfunctions of the body, like type 2 diabetes, atherosclerosis, and/or changes in lipid metabolism, are related to oxidative stress resulting from the generation of free radicals. Thus, antioxidative peptides naturally occurring in food sources are the focus of scientific interests [13].

Nowadays, it is almost impossible to avoid computer technologies when studying biopeptides [14]. According to Marvin et al. [15], it stems from the production of loads of data, which, due to its accessibility, brings new insights to the studied phenomena, improves decision-making, and enhances the quality of products and services. A consequence of quick data aggregation is the problem with its efficient collection, storage, and processing. To date, computer analyses have been applied for multiple purposes in this area, e.g., in silico analyses were deployed to study quantitative structure-activity relationships of milk protein-derived peptides with ACE-inhibiting [16] and DPP-IV-inhibiting [17] effects; the molecular docking of selected peptides identified in fermented milks of different animal species to study peptide inhibition against pancreatic α-amylase [18], peptidic ACE-inhibitory and DPP-IV-inhibitory effects, as well as the antioxidative activity of Gouda cheese with a modified content of β-casein [19,20]; and to predict the bitterness of milk protein hydrolysates resulting from the presence of peptide motifs known for their bitter-tasting properties [21]. All the above-mentioned studies required elaboration of the list of peptides with the above-mentioned activities. The information on peptides was acquired from the literature [16,17] or computer databases of molecules, including peptides [18,19,20,21]. According to Peredo-Lovillo [22], the latter are tools that facilitate screening peptide sequences for further analyses. Moreover, peptide databases play an important role in the development of bioinformatic (in silico) protocols enabling the characterization of biopeptides in terms of predicting, e.g.: (1) their protein sources, (2) their enzymatic release from proteins using simulated proteolysis, (3) their allergenicity and toxicity, and (4) their bioactivity despite their origin and targeted bioactivity [22]. Finally, the combination of in silico and in vitro protocols is a contemporary approach when studying food protein-derived biopeptides [14].

According to Kiełczewska et al. [23], milk protein-based preparations possess specific properties like solubility, dispersibility, water binding, foaming, whipping, emulsification, gelation, and buffering capacity. Thus, they can be used to modify the functionality of dairy foods in terms of their heat stability, viscosity, texture, and sensory value [23]. The addition of milk protein preparations to dairy products may also improve their digestibility [24]. For example, it was found that kefirs produced with the addition of different preparations (i.e., milk protein concentrate, whey protein concentrate, sweet whey, non-demineralized or demineralized whey, whey permeate, rennet casein, buttermilk) had an increased concentration of free lysine which serves as an indicator of protein digestibility [24]. Bioactivity is another feature that makes milk protein preparations value-added ingredients [25]. The research in this matter mainly concerned the enzymatic modifications of whey protein concentrates (WPCs) to produce a bifunctional, i.e., ACE-inhibitory, and antioxidative hydrolysate [26]. Additionally, the potential of WPCs was analyzed to find out which enzyme was the most useful in imparting the strongest ACE-inhibiting and antioxidative effect [25].

Although milk proteins are well-recognized sources of bioactive peptides [2], few articles can only be found on the production of milk protein-derived biopeptides using the INFOGEST standardized protocol, i.e., a procedure that simulates the gastrointestinal tract digestion of food components [27,28]. One of the relatively new works is a paper by Atallah et al. [29] who applied INFOGEST to digest six milk protein matrices subjected to industrial processes, followed by biochemical characterization of products, including peptide identification [29]. Our search in the Web of Science database (as of June 2024) [30] using the following words as queries, “milk proteins INFOGEST”, “milk protein preparations INFOGEST”, “in silico milk proteins INFOGEST”, and “milk proteins bioactive peptides INFOGEST”, revealed 57, 6, 0, and 9 records, respectively. Among them, there were no records related to the analysis of digested milk protein-derived preparations as sources of biopeptides.

Thus, taking into account the biopeptidic potential of milk proteins as well as the technological and nutritional value of milk-protein preparations, the aim of this study was to deploy in silico and in vitro protocols for the analysis of newly developed milk protein preparations and their hydrolysates as sources of selected enzyme inhibitors (i.e., against ACE and DPP-IV) and antioxidative peptides.

## 2. Results and Discussion

### 2.1. Bovine Milk Proteins as the Sources of Biopeptides—An In Silico Analysis Results

To date, bioactive peptides have been identified in various food protein sources, and enzymatic hydrolysis has been claimed to be one of the viable methods for producing biopeptides from foods [31]. Thus, the first step of the present study involved computer simulation of the hydrolysis of milk protein sequences using pepsin, trypsin, and chymotrypsin. It led to the release of 59 peptides (52 di- and 7 tripeptides), exhibiting both the ACE-inhibiting, DPP-IV-inhibiting as well as antioxidative effects (see Table S1 in the Supplementary Materials). One of the important aspects of the digestion of milk proteins in humans and animals is the absorption of generated bioactive peptides [32]. Thousands of di- and tripeptides, including their analogs, are absorbed into enterocytes (via Peptide Transporter 1, i.e., PEPT1 mechanism) and body organs, i.e., kidneys, lungs, mammary glands, the liver, and the brain (via PEPT2 mechanism). There are some studies indicating the presence of longer peptides in human plasma; however, it is still unknown if tetrapeptides or longer peptides can be transported into the circulation. Moreover, infants better absorb peptides than adults due to the immature brush-border membrane of their gastrointestinal tract. Thus, small peptides from milk and other foods play a more significant physiological role in infants than in adults [32]. The most abundant were ACE-inhibitory and DPP-IV inhibitory peptides, whereas five sequences showed antioxidant activity. Several peptides displayed more than one bioactivity. For example, a VW sequence exhibited all bioactivities listed in Table S1. According to scientific reports, ACE and DPP-IV inhibitors are the most abundant of the peptides, featuring various bioactivities, which translate into the results of computer predictions. A higher number of peptides with specific bioactivities found in the database is associated with a higher probability of them being released from proteins [19]. There is also another regularity, namely, the shorter the peptide sequence is, the greater is the probability it will be produced via protein hydrolysis, which was confirmed in our previous studies entailing combined in silico and in vitro protocols (i.e., a hybrid approach) [19,20,33]. It is worth mentioning that the bias resulting from a high number of DPP-IV and ACE inhibitors among the known bioactive peptides affects computer predictions but not experimental assays concerning entire hydrolysates. It is possible to determine the biological activity of a hydrolysate without prior knowledge about the sequences of its active peptides. The above finding may be supported by the outcomes of an experiment conducted by Moreno-Mariscal and co-workers [34]. They performed measurements of the inhibition of angiotensin-converting enzyme and neprilysin (EC 3.4.24.11) by hydrolysates of chicken carcasses, followed by identification of peptides. Most of the identified dipeptides were previously known as ACE inhibitors, but none as neprilysin inhibitors. The last step of their experiment entailed the measurement of neprilysin and ACE inhibition by individual peptides and allowed the discovery of new neprilysin-inhibiting peptides.

The BIOPEP-UWM database (https://biochemia.uwm.edu.pl/biopep-uwm/) [35] application for the computer simulation of proteolysis produced qualitative and quantitative results. The first shows particular peptide sequences (including their bioactivity) that can be potentially released from proteins. The latter are the mathematical parameters (i.e., A_E,_ W, B_E_, V; see Methods). Their particular definitions and mathematical formulas were described by Minkiewicz et al. [36] and Iwaniak et al. [19] and are provided in the BIOPEP-UWM database (see the bar called “Definitions”). These parameters serve to analyze the potential effectiveness of enzymes in the biopeptide(s) release. Their values indicating the effectiveness of proteolysis of bovine milk proteins using pepsin, trypsin, and chymotrypsin in producing ACE and DPP-IV inhibitors are shown in Table 1.

The content of Table 1 lists proteins exhibiting two inhibitory activities, i.e., against ACE and DPP-IV. As mentioned above, it results from the number of peptides with specific activity in the BIOPEP-UWM database (the higher the number, the stronger the activity). The results were achieved for a small number of peptides other than ACE and DPP-IV inhibitors that were released from bovine milk proteins, which were impossible to interpret due to several reasons. Firstly, if a peptide is released from one protein only (out of several analyzed), it is not possible to establish which proteins are better/worse sources of peptides with specific bioactivities (based on A_E_). Secondly, parameters B_E_ and V suggest stronger/weaker activity of the released peptides and are calculated based on IC_50_ values. The lack of these values might cause understated results, or an IC_50_ that equals 0, which makes the calculations impossible (the null value in the denominator). For these reasons, the values of A_E_, W, B_E_, and V used to predict the effectiveness of the release of antioxidative peptides from all the bovine milk proteins analyzed could not be determined.

Based on the predictions (see Table 1), it can be said that DPP-IV inhibitors tend to be released from milk proteins more frequently than ACE inhibitors (A_E_ is higher for the DPP-IV inhibiting effect). Values of W suggest that the digestive enzymes applied are more effective in producing DPP-IV than ACE inhibitors. Taking into account A_E_ and W values, it seems that casein fractions tend to be better sources of peptidic ACE and DPP-IV inhibitors than whey and serum proteins. In turn, B_E_ and V values suggest relatively weak activity of the released DPP-IV inhibitors. However, it needs to be emphasized that the values of the B_E_ and V parameters might be understated. Their mathematical formulas require the values of IC_50_. If they are unknown (i.e., not quantified) for certain peptides, the BIOPEP-UWM application eliminates such peptides from the calculations, which affects the ultimate results [36]. Moreover, the BIOPEP-UWM application for the simulation of protein hydrolysis is simplified and does not include misscleavages. It assumes that all peptide bonds are broken during the proteolysis due to the specificity of the enzyme (or combination of enzymes). In practice, hydrolysis is incomplete, and a part of the peptidic bonds are not hydrolyzed. The discrepancies between computer predictions and wet-lab results were explained in our previous works [21,37,38,39]. However, despite incomplete hydrolysis in wet-lab conditions, the presence of biopeptides that had been theoretically released from their parent proteins was confirmed using mass spectrometry or high-performance liquid chromatography in, e.g., milk or cheese [19,21].

### 2.2. Progress in the Hydrolysis of Bovine Milk Protein Preparations (MPPs)

It is a common fact that proteins are metabolized in the body, leading to their defragmentation, which, in turn, produces peptides and free amino acids. Regardless of the environmental conditions (in vivo or in vitro), proteins are degraded due to the action of digestive enzymes (proteases) [40]. Thus, the next part of the experiment involved the simulated digestion of MPPs using the INFOGEST standardized protocol [27,28]. The progress in MPPs hydrolysis was monitored by calculating the protein content of the samples before (0 phase, i.e., start point) and after the final phase (D phase, i.e., endpoint) of INFOGEST digestion [28]. Respective results are shown in Table 2. According to Akimov and Bezuglov [41], the importance of digestion stages is not equivalent and depends on, e.g., toxicity, allergenicity, and/or nutritive value of a digested molecule. The bioactivity of the produced peptides can also be one of such factors when thinking about the importance of protein digestion stages. The main function of the stomach is to monitor the successive delivery of food into the small intestine, and it can be considered dispensable for overall protein absorption. Hence, the gastric phase is not essential because pepsin breaks protein into smaller segments to increase its availability for the other digestion steps. Therefore, the duodenal phase of protein digestion, leading to the production of di- and tripeptides, is more critical than the gastric phase. The activity of such peptides is protected due to the multiple isoforms of endopeptidases [41].

The protein content of the samples decreased as the hydrolysis progressed (see Table 2). The highest percentage of protein was determined in the samples before simulated digestion (i.e., 0 phase) and ranged from 53.67% (SPC) to 68.67% (MCC). After the duodenal phase of digestion (D phase), the protein content of each MPP decreased and ranged from 26.33 (MBP D) to 32.33% (MCC D).

These results were also the basis for monitoring changes in the protein and peptide profiles during the simulated digestion of the MPPs. Such changes were observed using reverse-phase high-performance liquid chromatography (RP-HPLC) [19,20]. Chromatography techniques, including RP-HPLC, are commonly used to monitor the progress of proteolysis in food materials [19,20,21,42,43], including hydrolysis of food proteins that imitates gastrointestinal tract digestion [42,43,44,45].

**Figure 1:**
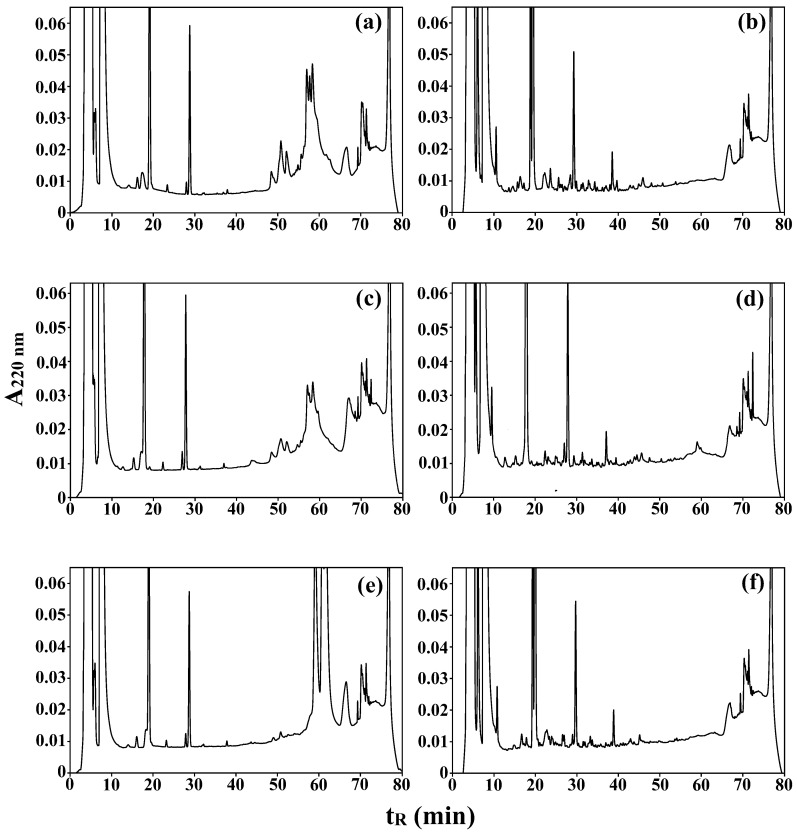
RP-HPLC chromatograms of: (**a**) micellar casein concentrate (MCC) before simulated digestion; (**b**) MCC subjected to the duodenal phase of simulated digestion (MCC D); (**c**) MCC with ultrafiltrated buttermilk permeate (MBP) before simulated digestion; (**d**) MBP subjected to the duodenal phase of simulated digestion (MBP D); (**e**) serum protein concentrate (SPC) before simulated digestion; and (**f**) SPC subjected to the duodenal phase of simulated digestion (SPC D); t_R_ (min)—retention time (min).

Figure 1a–f shows chromatograms of three bovine milk protein preparations before (Figure 1a,c,f) and after the final phase (i.e., D phase) of simulated digestion (Figure 1b,d,f).

Total peak areas calculated for the following retention time segments [min]: 0.00–9.99, 10.00–19.99, 20.00–54.99, and 55.00–70.00 are shown in Table 3. The last fraction corresponds to non-hydrolyzed proteins. The main milk proteins were eluted in the following order: κ-casein, α_s2_-casein, α_s1_-casein, β-casein, β-lactoglobulin, and α-lactoalbumin [45,46]. κ-Casein was probably eluted before the 55th minute (Figure 1a,c,e). Thus, the 55th minute of chromatographic separation was found as the retention time of the last peaks corresponding to the simulated digestion products.

Analysis of chromatograms was focused on changes in the areas of peaks within time intervals. The components of individual peaks were not identified. Apart from proteins and peptides, other compounds, e.g., bile salts or bile acids, were likely to appear. Such chromatograms show the progress in proteolysis, especially a decrease in the content of the fractions with the longest retention times (containing proteins) and an increase in the content of the fractions with retention times within the ranges of 10–19.99 and 20–54.99 min. A lack of identification of individual components does not make this goal impossible to achieve. The highest peak area was observed for the proteins that were eluted in the first retention time segment (0.00–9.99 min), and may deemed the so-called injection peak indicating material that is not adsorbed on the column, e.g., low-molecular mass compounds found in MPPs, components of the solutions that were used for sample dissolution [46] and/or the components of buffers used for the particular phase of the INFOGEST standardized digestion protocol [28]. Injection peaks are also typical of short peptides, being the products of protein hydrolysis [19]. No repeatable relationships between the type of MPP and the relative peak area corresponding to retention time before the 10th minute, that could definitely confirm the presence of proteolytic products as well as the changes in their content, were observed in this part of experiment despite statistically significant differences found between the relative peak areas of compounds eluted in this retention time segment (see Table 3). Moreover, it can be expected that the percentage of protein fractions eluted before the 10th min will increase as the hydrolysis continues.

Peaks observed between the 70th and 80th minutes were not interpreted. They are defined as system peaks [47], which are artifacts that do not provide information on the sample; however, they hinder the interpretation of chromatograms. The reason for this is the overlapping of system peaks with sample components having long retention times. Thus, system peaks were not taken into account in this experiment. Although this peak occurs in all chromatograms, its area varies. It is noticeable especially when comparing Figure 1c with Figure 1d and Figure 1e with Figure 1f. It is likely that some proteins from the undigested samples coeluted with the system peak at 68th min and disappeared after digestion.

All MPP samples subjected to duodenal simulated digestion (D phase) contained more biological material eluted between 10.00 and 19.99 min than their respective MPPs in the earlier phases of digestion, which suggests the presence of final products of MPPs hydrolysis in D phase. The content of the fraction eluted between 50.00 and 70.00 min was the same for all MPP samples subjected to phase D of simulated digestion. A peak that appeared between the 68th and 69th min in all chromatograms (see Figure 1) was probably a system peak [47] which was included in our calculations.

To recapitulate, considerable progress in the proteolysis, expressed as the increase in the content of peptides eluted between 10.00 and 19.99 min, was observed in all MPPs subjected to phase D when compared with MPPs before simulated digestion. During phase D of skim milk digestion, such an increase was observed for the content of the fraction eluted between 20.00 and 54.99 min. Szymczak et al. [48] found that an increase in the content of the fraction with shorter retention times corresponded to an increase in the content of the fraction with lower molecular masses, measured using mass spectrometry in brine after herring marination. The conclusion from the above-cited experiment may also be applied to the present work.

Thus, the changes observed in the protein and peptide profiles of MPPs during the simulated digestion formed grounds for the next stage of the experiment, i.e., determination of bioactivity of MPPs and their respective hydrolysates, which included the following assays: inhibition of ACE and DPP-IV as well as antioxidative activity.

### 2.3. Bioactivity of MPPs and Their Respective Hydrolysates

Table 4 presents data from determinations of the bioactivity of bovine milk protein preparations before and after the final phase (phase D) of simulated digestion. The bioactivity of such a sample could indicate its potential as, e.g., an additive for functional food production. The bioactivities listed in Table 4 were expressed as IC_50_ (i.e., the concentration of the sample corresponding to its half-maximal bioactivity; mg/mL). The conversion of antioxidant activity to IC_50_ was applied by Iwaniak et al. [20] to measure the antioxidative effect of Gouda cheese variants with different β-casein contents, and is in line with Dontha [49], according to whom “an antioxidant is a molecule capable of inhibiting the oxidation of another molecule”.

Regardless of the digestion phase, almost all MPPs showed the bioactivities in Table 4. An exception was MCC, which IC_50_ against DPP-IV was not detectable. Among all non-hydrolyzed samples, MCC showed the highest activity against ACE (IC_50_ = 1.856 mg/mL), whereas SPC exhibited the lowest antioxidative power (IC_50_ = 3.738 mg/mL). In the case of digested MPPs, the most ACE-inhibiting potential had MCC D (IC_50_ = 2.897 mg/mL). In turn, MBP D (IC_50_ = 7.627 mg/mL) exhibited the weakest ACE inhibition. It was observed that MCC possessed the highest ACE inhibitory bioactivity among all samples (finally digested or not).

Although the potential of MCC to inhibit DPP-IV was not detectable (see Table 4), its hydrolysate (MCC D) exhibited this activity (IC_50_ = 0.404 mg/mL). Among the non-hydrolyzed MPPs, the highest ability to inhibit DPP-IV was determined for MBP (IC_50_ = 0.561 mg/mL). MBP D showed the best DPP-IV-inhibiting potential among all hydrolyzed samples (IC_50_ = 0.0067 mg/mL). Among the analyzed samples (before and after the final phase of digestion), SPC and SPC D exhibited the lowest DPP-IV inhibition (IC_50_ = 1.693 and 0.512 mg/mL, respectively).

Regardless of the assay and digestion phase, all MPPs had antioxidative power. MPPs after phase D of simulated digestion had a higher antioxidative power (all three tests) than their analogical samples before digestion, except MCC D (IC_50_ = 4.673 mg/mL). Taking into account the results using ABTS^•+^, MBP D was the MPP with the highest antioxidative potential (IC_50_ = 2.754 mg/mL). The lowest antioxidative effect was found for SPC before hydrolysis (IC_50_ = 6.244 mg/mL). The antioxidative assay using DPPH^•^ revealed that SPC D had the highest radical scavenging power (IC_50_ = 1.238 mg/mL). Among all samples, the lowest DPPH^•^ scavenging bioactivity was determined for MBP D (IC_50_ = 1.687 mg/mL). In turn, the FRAP analysis revealed that all samples after the final phase of digestion (phase D) had a higher antioxidative power than their analogical non-digested preparations. Among them, the strongest FRAP potential was shown for SPC D (IC_50_ = 13.720 mg/mL), and the weakest for MBP D (IC_50_ = 20.140 mg/mL). A similar observation was made for the non-digested SPC and MBP—their FRAP was the strongest for SPC (IC_50_ = 14.470 mg/mL) and the weakest for MBP (IC_50_ = 31.120 mg/mL).

The antioxidant power of MPP and its hydrolysates was measured using three tests involving ABTS, DPPH, and FRAP. To date, there is no normalized method to determine the antioxidant power of foods. Thus, it is recommended to apply tests that are based on different mechanisms [50,51], like HAT (hydrogen atom transfer) and SET (single electron transfer). The ABTS method falls under the HAT mechanism, whereas DPPH and FRAP tests are based on the SET mechanism [52]. HAT and SET reactions may occur simultaneously, as affected by the structure of the antioxidative compound, its solubility, n-octanol/water partition coefficient, and polarity of the solvent [52]. The issues related to the quantification of antioxidant power, as well as its pros and cons, were discussed by Iwaniak et al. [20]. All of these factors may explain, e.g., the different order of magnitude and high/low activity measured for the same sample using different assays. Moreover, the antioxidant power of the biological material may result from the location of a peptide in a protein structure, its hydrophobicity, methods applied for protein isolation, degree of protein hydrolysis, enzymes applied, protein concentration in a substrate, and amino acid configuration in a peptide chain [13]. These factors are also applicable to the results of inhibiting bioactivities quantified in the present study.

Regardless of the bioactivity assay (see Table 4), some results showed that the activity of certain MPPs decreased after the final phase of sample digestion compared to the non-digested samples. This observation referred to the ACE inhibitory activity of all MPPs (they had a higher activity than their digested samples), ABTS^•+^ radical scavenging effect of MCC, DPPH^•^ radical scavenging, as well as the FRAP effect of MBP. The suppression of MPP bioactivity after the final phase of digestion, when compared to its non-digested sample, may suggest that products before digestion contain some compounds responsible for bioactivity and, at the same time, are susceptible to digestion. The phenomenon of hydrolysis of bioactive peptides by gastrointestinal enzymes, resulting in the loss of activity, has been addressed earlier in the literature [53,54,55].

The INFOGEST protocol, applied to simulate substrate (like MPP) digestion in a body, revealed the discrepancies between the samples and their biological effects at different phases of hydrolysis. Such results show the complexity of the digestion process. Depending on its phase, the released peptides might be degraded to, e.g., amino acids, which may affect the suppression or loss of bioactivity. In turn, the high bioactive power may result from a synergistic effect of bioactive agents of a hydrolysate. Another issue is the peptides’ stability—they are easily degraded in the gastrointestinal tract; therefore, to deliver them in an active form requires undertaking some additional technological operations, like, e.g., encapsulation [56].

To summarize, the bioactivity of all samples (digested or not), referring to ACE and DPP-IV inhibition as well as their antioxidative effects, may result from the presence of biopeptides. Thus, the final step of the present study aimed to identify them in the MPPs and their hydrolysates.

### 2.4. Identification of Biopeptides in MPPs and Their Respective Hydrolysates

In silico hydrolysis of bovine milk proteins, using the BIOPEP-UWM module [36] showed that the simultaneous action of pepsin, trypsin, and chymotrypsin led to the release of 58 bioactive peptide sequences. Thirty-six of them were identified in at least one of the three MPP samples (i.e., MCC, MBP, and SPC) and subjected to simulated digestion using the INFOGEST protocol [28]. The results of the identification of these peptides are collated in Table 5. They include peptides found in MPPs at the initial and final phases of simulated digestion (see the explanation in the above Sections). It was observed that MPPs before digestion contained from one to four bioactive peptides. For example, a peptide with an SG sequence was identified in MPPs before digestion, but it was not detected in the digested samples. Some peptides were identified in both the non-digested and digested MPPs, like, e.g., VA (present in: MCC, MCC D, MBP, MBP D) vs. (present in: MCC, MCC D, MBP, MBP D), and VP (present in: MBP, MBP D). Some of the identified peptides contained methionine residues. They may be oxidized to methionine sulfoxide. Enzyme inhibition or other biological activity of such peptides is usually measured using peptides containing methionine, and not methionine sulfoxide. The BIOPEP-UWM database includes 689 peptides containing methionine (as of April 2025). Only two of them contain oxidized methionine. Regarding the peptides listed in Table 5, there are no data about the activity of their forms with oxidized methionine.

The differences in retention times obtained for the same peptide ranged from 0 to 10 s (see Table 5), except for VPL (15 s) and IPM (56.4 s). Such differences in retention times identified for the same peptides using LC-MS and acetonitrile (i.e., ACN), water, and formic acid as the mobile phase are typical and were described elsewhere [57,58,59]. Among the 36 peptides identified in at least one of the MPPs, the retention times of 30 of them were below 5 min, and for 6 of them, the times ranged from 5 to 10 min. Only two peptides had retention times above 10 min. Although different columns, as well as the composition of the mobile phase, were applied in this experiment, it may be speculated that the majority of peptides—the final products of hydrolysis—were probably eluted in the first fraction during RP-HPLC experiment (using trifluoroacetic acid, i.e., TFA, as mobile phase). Fractions with longer retention times probably contained the longer-chain peptides [19,21,39].

**Figure 2:**
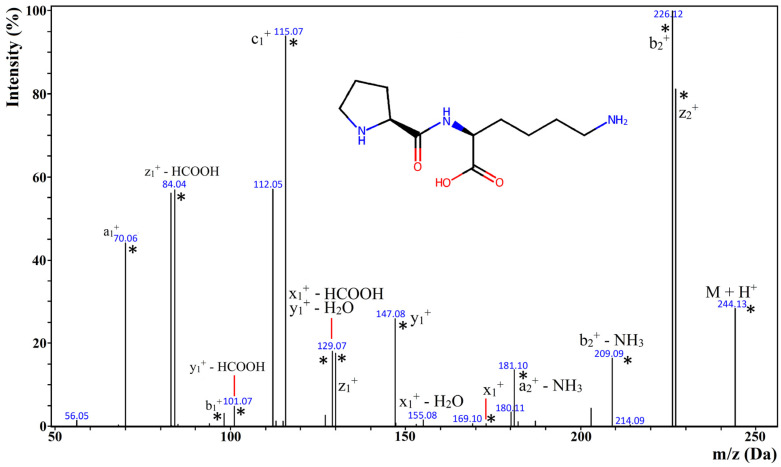
MS/MS spectrum of a PK sequence. Fragmentation ions were systematized according to the nomenclature introduced by Roepstorff and Fohlman [60]. Asterisks denote the fragmentation ions present in the reference spectrum of the same peptide found in the METLIN database [65,66].

The tandem mass spectroscopy (i.e., MS/MS) spectra of the identified peptides contained typical fragmentation ions, i.e., a, b, c, x, y, and z [60,61,62]. There could also be observed ions generated from neutral loss (i.e., release of molecules such as water, ammonia, and formic acid). They are considered the typical ions produced during the fragmentation of peptides [60,62]. The release of formic acid was often observed during tandem mass spectrometry of dipeptides [63]. In some cases, the fragmentation ion mass could be associated with more than one chemical structure. An example of such an ion is shown in Figure 2, which presents the MS/MS spectrum of PK peptide identified as the product of duodenal digestion of MCC, MBP, and SPC. According to Lan et al. [64], PK was confirmed as a DPP-IV inhibitor (BIOPEP-UWM ID: 8858). The fragmentation ion corresponding to more than one chemical structure referred to the peak associated with 129.07 Da and the following ions: x_1_^+^—HCOOH and y_1_^+^—H_2_O. They are both isomers. In turn, the spectra of two isomeric peptides, i.e., PL and IP, which were eluted within the same time, contained fragment ions characteristic of both sequences (see Figure S1 in the Supplementary Materials).

Depending on the MPP, the number of identified peptides ranged from 28 to 30 (see Table 6). The discrepancies between in silico results of BIOPEP-UWM-assisted hydrolysis (59 peptides predicted to be released; Table S1 in the Supplementary Materials) of bovine milk proteins and simulated digestion of bovine milk protein preparations (28–30 peptides released) could be explained by the incomplete hydrolysis of milk proteins which was mentioned when discussing computer prediction analyses. Thirty peptides were identified in the casein-containing preparations, i.e., MCC and MBP, after duodenal digestion, and 28 were found in the whey protein-containing sample (SPC D). Twenty-three peptides were identified in the digested samples of all MPPs, whereas five sequences (PT, SE, IN, PR, and IW) were present only in the SPC (see Table 5).

Di- and tripeptides were identified in all digested milk protein preparations derived from all casein fractions and/or major whey proteins. Short peptides, like di- and tripeptides, occurring in the sequences of many proteins, can be found via in silico sequence matching and detected experimentally [33]. An example of a peptide derived from casein is VPL (source: α_s1_-casein) found in SPC subjected to digestion (i.e., SPC D). In turn, IPA (source: β-lactoglobulin) and IPM (source: lactoferrin) were detected in MCC D (IPA) and duodenally digested MPPs (IPM). It needs to be explained that ultrafiltration was applied to produce all MPPs. According to scientific reports, it does not lead to the complete separation of casein from whey proteins [67,68].

It is impossible to attribute di- and tripeptides to an individual protein sequence [69]. Only 10 peptides detected in this experiment (PK, PM, VM, PH, IE, IH, IPA, IW, PPL, and IPM) were absent in sequences of pepsin, trypsin, and chymotrypsin (IDs: P00761, P00791, and P00766 in the UniProt database (as of April 2025). They may be released only from milk proteins. It is, however, likely that other peptides may be released during the autolysis of proteolytic enzymes. Data from Table 5 suggest that peptides were released from milk proteins and not from enzymes. The enzyme-derived peptides could be expected in all samples after the duodenal phase of digestion. Eleven peptides (PT, VT, VL, SK, IN, PR, VR, IR, IPA, IW, and PPL) occurred only in part of the samples. Only the last three of them were absent in sequences of enzymes. The same enzyme preparations and the same conditions were applied for the simulated digestion of all samples of milk proteins. The same pattern of autolysis should thus be expected. Various technological processes were applied to produce particular protein preparations that differed [70,71,72], which could have resulted in different susceptibility of individual proteins to enzymatic hydrolysis. An example of differences in the proteolytic pattern of the same protein isolated using different processes (ultrafiltration, ion exchange, acid precipitation, or microfiltration) was described for alfalfa RuBisCo by Tanambell and co-workers [73]. A detailed molecular mechanism of the above phenomena has not been described to date, although different treatments of protein have led to changes in its supramolecular structure [74].

Biological activities of the released peptides found in all bovine milk preparations subjected to simulated digestion are shown in Table S1 (see the Supplementary Materials), whereas their quantitative characteristics, understood as the numbers of biopeptides representing individual activities, are provided in Table 6. Information in Table S1 is based on data from the BIOPEP-UWM database and includes the activities being the subject of this article, as well as the IC_50_ values [μM] of the identified peptides (if any).

DPP-IV and ACE inhibitory effects were found to be the dominant bioactivities of the identified peptides. A similar observation was made during computer predictions, being the effect of a positive selection approach (see the chapter above). Twenty-seven DPP-IV inhibitors were identified in MBP D and MCC D, and twenty-three in SPC D (see Table 6). Ten (MCC D) to eleven (MBP D and SPC D) peptides were found to exert the ACE inhibitory effect. Antioxidative activity was exhibited by a maximum of three peptides identified in MCC D and MBP D.

To recapitulate, among the 36 peptides that were identified in the duodenally digested MPPs (see Table S1), 11 sequences possessed dual- or triple-functionality (two peptides showed triple-bioactivity, nine—double). Most of the dually acting peptides displayed the ACE and DPP-IV inhibiting potential. Dipeptides with IR and VY sequences showed three activities, which are the subject of our studies. Five peptides (IE, IG, PR, SG, ST) displayed only ACE inhibitory effect. Thirteen ACE-inhibiting peptides (IG, IP, IR, IW, PL, PR, SG, ST, VG, VP, VR, VY, IPA) had the IC_50_ value (see Table S1). For four of them (IW, PR, ST, and VY), the IC_50_ was below 10 µM. IW and PR were identified in SPC D, whereas ST and VY were identified in all the three duodenally digested preparations. Among DPP-IV inhibitors, 10 of them were characterized by the IC_50_ parameter. The strongest inhibitors (taking into account IC_50_) were VL, IPA, IPM, and VPL, with their IC_50_ ranging from 15.80 to 74.00 µM. Among them, VL and IPM peptides were identified in all digested MPP samples. In turn, IPA and PPL were detected in digested casein-containing milk protein preparations, i.e., MCC D and MBP D. As it could be observed, only di—and tripeptides were identified in this experiment. Other authors identified longer sequences in their experiments concerning the digestion of milk proteins in the gastrointestinal tract [75,76,77,78,79]. Although RP-HPLC-UV results suggested that casein was less susceptible to proteolysis than whey proteins, more bioactive peptides were identified in the casein-containing milk protein preparations (MCC and MBP) than in the whey-containing one (SPC).

## 3. Materials and Methods

### 3.1. Materials

The following reagents were purchased from Sigma-Aldrich Sp. z o. o., Poznań, Poland: pepsin from porcine gastric mucosa (EC 3.4.23.1, No. P7012, ≥2500 units/mg protein), pancreatin from porcine pancreas (No. P7545, 8 × USP specifications), porcine bile extract (No. B8631), ACE from rabbit lung (EC 3.4.15.1, No. A6778, ≥2.0 units/mg protein), bovine serum albumin (BSA)—protein standard (No. P0834), recombinant human DPP-IV (EC 3.4.14.5, No. D3446, ≥4500 units/µg protein), trifluoroacetic acid (TFA, No. T6508-25ML-D), urea (catalog No. U5378), 2,2-bis(hydroxymethyl)-2,2′,2″-nitrilotriethanol (BIS-TRIS, No. B9754), 2-mercaptoethanol (No. M3148-100ML), borate buffer (No. 82634), hippuryl-histidyl-leucine (HHL; No. H4884), Gly-Pro-*p*-nitroanilide (Gly-Pro-*p*-NA; No. G2901), TRIS(hydroxymethyl)aminomethane hydrochloride (TRIS-HCl; No. 93313), 2,2-diphenyl-*β*-picrylhydrazyl (DPPH, No. D9132), 2,2′-azino-bis(3-ethylbenzotialozline-6-sulfonic acid (ABTS, No. A3219), phosphate-buffer saline solution (PBS, No. P4417), pyridine (No. 270970), Gly-Pro *p*-nitroanilide hydrochloride (No. G0513), ferrozine (TPTZ, 2,4,6-Tris(2-pyridyl)-s-triazine, No. T1253), ferric chloride hexahydrate (FeCl_3_ × 6H_2_O, No. 236489), Folin–Ciocâlteu reagent (No. 1.09001), and DL-dithiothreitol (DTT; No. D0632).

Reagents for MS/MS analyses were purchased from Merck, Poznań, Poland (LiChrosolv^®^ and LiChropur^®^) and used without further purification. They were as follows: water for chromatography (LC-MS Grade; No. 1.15333), acetonitrile hypergrade (No. 1.00029), and formic acid (No. 5.33002).

Other chemical reagents used for analyses were supplied by Chempur (Piekary Śląskie, Poland): hydrochloric acid, sodium hydroxide, glacial acetic acid, potassium chloride, potassium phosphate, dipotassium phosphate, sodium bicarbonate, sodium chloride, magnesium chloride, ammonium carbonate, calcium chloride, and copper (II) sulfate.

Water used to formulate solutions and buffers was prepared using a Milli-Q PLUS system (Millipore Corp., New York, NY, USA). Nylon membrane filters (Whatman^®^, 0.2 μm pore size, catalog No. WHA7402004) and Amicon^®^ Ultra Centrifugal Filters were purchased from Sigma-Aldrich Sp. z o.o. (Poznań, Poland), and Munktell-Filtrak 390 grade filters (catalog No. 8.012.120.900) from EQUIMED (Olsztyn, Poland).

All chemicals and reagents were of analytical and/or MS grade.

### 3.2. Methods

#### 3.2.1. In Silico Analysis of Bovine Milk Protein Sequences for Enzymatic Release of Selected Enzyme Inhibitors and Antioxidative Peptides

The following sequences of bovine (*Bos taurus*) milk proteins were acquired from UniProtKB (www.uniprot.org/) for in silico hydrolysis [80,81]: α_s1_-casein, genetic variant B (i.e., α_s1_-CN, B; P02662/BIOPEP-UWM ID: 1087/199 aa); α_s2_-casein, genetic variant A (i.e., α_s2_-CN, A; P02663/BIOPEP-UWM ID: 1090/222 aa); β-casein, genetic variant A1 (i.e., β-CN, A1; P02666/BIOPEP-UWM ID: 1097/209 aa); κ-casein, genetic variant A (i.e., κ-CN, A; P02668/BIOPEP-UWM ID: 1117/190 aa); α-lactalbumin, genetic variant A (i.e., α-La, A; P00711/BIOPEP-UWM ID: 1831/123 aa); β-lactoglobulin, genetic variant A (i.e., β-Lg, A; P02754/BIOPEP-UWM ID: 1821/161 aa); bovine serum albumin (i.e., BSA; P02769/BIOPEP-UWM ID: 1729/607 aa); and lactoferrin (i.e., Lf; P024627/BIOPEP-UWM ID: 1236/708 aa). Data in brackets refer to the abbreviation of the protein, accession number in SwissProt, being part of UniProt database, BIOPEP-UWM database ID, and the number of amino acids, respectively. For example, α_s1_-CN, B; P02662/BIOPEP-UWM ID: 1087/199 aa should be understood as α_s1_-casein, genetic variant B, where P02662 is the SwissProt/UniProt accession number; BIOPEP-UWM ID: 1087 is the ID number of this protein in the BIOPEP-UWM database, and 199 aa is the number of amino acids the protein is composed of. The above-mentioned sequences correspond to the genetic variants of these proteins that were most frequently present in the milk of Friesian-Holstein cows [82].

The hydrolysis of milk protein sequences was simulated by means of a BIOPEP-UWM database–associated tool called “Enzyme(s) Action” using the combined action of pepsin (EC 3.4.23.1; BIOPEP-UWM-ID: 39), trypsin (EC 3.4.21.4; BIOPEP-UWM-ID: 12), and chymotrypsin (EC 3.4.21.1; BIOPEP-UWM-ID: 11) [35]. The specificity for pepsin referred to that corresponding to pH > 2.0 to maintain the consistency with the simulated digestion procedure introduced by Brodkorb et al. [28]. The effectiveness of proteolysis of bovine milk proteins using pepsin, trypsin, and chymotrypsin in producing ACE and DPP-IV inhibitors was calculated using parameters defined as the frequency of release of fragments with a given activity by selected enzymes (A_E_), the relative frequency of release of fragments with a given activity by selected enzymes (W), activity of fragments potentially released by proteolytic enzyme (enzymes) (B_E_), and relative activity of fragments potentially released by proteolytic enzyme (enzymes) (V). Their mathematical formula were characterized by Minkiewicz et al. [36].

All computer-assisted predictions were carried out from May to September 2024.

#### 3.2.2. Laboratory Analyses of Bovine Milk Protein Preparations (MPPs) Laboratory Analyses of Bovine Milk Protein Preparations (MPPs)

##### Milk Protein Preparations (MPPs)

Three milk protein preparations, namely: MCC (micellar casein concentrate), SPC (serum protein concentrate), and MBP (MCC with ultrafiltrated buttermilk permeate), were produced in a pilot-plant scale at the Department of Dairy Science and Quality Management of the University of Warmia and Mazury in Olsztyn, Poland [70,71,72].

##### Simulation of Human Digestion of MPPs Using the INFOGEST Protocol

*General information:* The INFOGEST in vitro harmonized static model introduced by Minekus et al. [27] and Brodkorb et al. [28] was applied to simulate the human gastrointestinal digestion of milk protein preparations (MPPs). To this end, three types of samples (5 mL each) were prepared to simulate the following phases of digestion: undigested (i.e., intact), gastric, and duodenal. They were abbreviated as 0, G, and D, respectively. According to Minekus et al. (2014), a dose of pepsin that was used in samples G and D was set to correspond to 2000 U/mL of a digestive fluid. In turn, a dose of pancreatin that was used in sample D corresponded to 100 U of trypsin/mL of a digestive fluid, whereas a dose of a bile salt extract corresponded to 4000 U/mL of a digestive fluid [27]. After each phase of simulated digestion, the samples were stirred and incubated at 37 °C using a KL-942 rocking platform shaker (RockingShaker, OHAUS Europe GmbH, Nänikon, Switzerland).

*Brief description of simulated digestion:* At the first phase of digestion, 5 mL of a fluid mimicking the human saliva was added to samples 0, G, and D. After 2 min of incubation, sample 0 was set aside. Then, 10 mL of a gastric fluid with pepsin was added to samples D and G. The duration of phase G was 60 min, and afterwards, the pH of the sample was adjusted from 3.0 to 7.0 by adding 1.0 M NaOH, and sample G was collected. The next step involved adding 20 mL of a duodenal fluid with pancreatin and bile salts to sample D. After 120 min of incubation, the samples were inactivated at 100 °C (5 min) and described as “duodenal-phase digests”. In vitro digestion was carried out in triplicate. Respective hydrolysates (digests) derived from each repetition were combined and lyophilized (Beta 1–8 LSCbasic, CHRIST, Osterode am Harz, Germany), and then stored at −80 °C until analyzed.

##### Protein Content

The protein content of the samples was quantified according to the Lowry method [83], which was based on the standard plot made for BSA (bovine serum albumin) and its concentration ranging from 0.1 to 1.0 mg/mL. Briefly, 2.0 mL of a standard solution or a sample was intensively mixed with 1.0 mL of an alkaline copper solution (CuSO_4_; prepared 30 min before the assay). Then, the samples were incubated at room temperature (10 min). After incubation, 0.1 mL of the Folin–Ciocâlteu reagent (FCR) was added to a sample, and the mixture was intensively stirred. After 30 min, sample absorbance was measured at 750 nm (GENESYS 150 Spectrophotometer, Thermo Scientific, Waltham, MA, USA). Based on the results, a standard curve—being the function of absorbance and BSA concentration—was plotted (Excel, Microsoft Office 2010). The protein content of the sample was read from the curve. The assay was performed in triplicate, and the results (see Section 2) are presented as mean values.

##### The Monitoring of MPPs Hydrolysis Processes by Means of Reversed-Phase High-Performance Liquid Chromatography with UV-Visible Spectroscopy (RP-HPLC UV/Vis)

Observations of changes in the peptide profiles during the simulated digestion of MPPs were carried out using reversed-phase high-performance liquid chromatography with UV-visible spectroscopy (RP-HPLC UV/Vis) [19]. Analysis was carried out using the Shimadzu^®^ system (Tokyo, Japan), which comprises two LC-20AD pumps, an SIL-20AC HT autosampler, a CBM-20A controller, a CTO-10AS VP thermostat, an SPD-M20A photodiode detector, a DGU-20A5 degasser, and an FRC-10A fraction collector. The Jupiter Proteo Phenomenex^®^ column (Torrance, CA, USA; 250 × 2 mm; particle diameter: 4 μm; pore diameter: 90 Å) was applied for RP-HPLC analysis of the samples. Solvent A was a 0.01% TFA (i.e., trifuloroacetic acid) water solution (*v*/*v*), whereas solvent B was 0.01% (*v*/*v*) TFA dissolved in acetonitrile (ACN) [19]. The gradients of solvents A and B used in chromatographic analysis of the samples are shown in Table 7.

MPPs and their respective hydrolysates were prepared as follows: 1 mg of a freeze-dried sample was dissolved in 400 μL of a buffer (pH = 6.6) containing 0.1 M BIS-TRIS and 4.0 M urea. Then, 20 μL of 2-mercaptoethanol was added to the mixture, which was vortexed and incubated (1 h, room temperature). After the incubation, 700 μL of a buffer solution in a mixture of acetonitrile and water at a ratio of 100:900 (*v*/*w*; pH 2.2 adjusted by TFA addition) was added to the sample and stirred. Finally, the sample was centrifuged at 10,000 rpm for 10 min [44] and moved to Chromacol test tubes. The temperature of the column was 30 °C, the flow rate was 0.2 mL/min, and the injection volume was 20 μL. Data were registered between minutes 0 and 80 at λ = 220 nm and then analyzed using the Shimadzu^®^ Lab Solution (LC Solution) program.

##### Bioactivities of MPPs and Their Hydrolysates

(1) ACE inhibition

The ability of MPPs and their respective hydrolysates against ACE inhibition and their respective hydrolysates was assayed using the spectrophotometric method introduced by Jimsheena and Gowda [84] with slight modifications proposed by Darewicz et al. [37]. Such an activity was measured in microplates at 37 °C in a mixture consisting of 35 μL of a 5 mM solution of HHL (hippuryl-histidyl-leucine) in 0.05 M borate buffer (pH 8.2) containing 0.3 M NaCl, 7.5 μL of an ACE solution (100 mU/mL) pre-incubated for 10 min with 7.5 μL of MPPs dissolved in a borate buffer. After 30 min, the reaction was stopped by the addition of 50 μL of a 1 M HCl solution. Next, 100 μL of pyridine and 50 μL of BSC were added sequentially to the samples. The samples were mixed very carefully and then cooled on ice. Absorbance was measured at a wavelength of 410 nm using a microplate reader (iMark Microplate Absorbance Reader; Bio-Rad, Hercules, CA, USA).

The percentage of ACE inhibition was calculated using Equation (1):

% _ACE inhibition_ = [(A_1_ − A_2_)/(A_1_ − A_3_)] × 100 (1)

where A_1_—absorbance of the mixture containing buffer instead of a potential inhibitor; A_2_—absorbance of the mixture containing the potential inhibitor, i.e., sample (MPP or its respective digest); and A_3_—absorbance of the mixture containing buffer instead of an enzyme solution and a potential inhibitor [85]. The assay was carried out in duplicate for the 5 concentrations of the samples, ranging between 1.0 and 10 mg/mL.

(2) DPP-IV inhibition

Inhibitory activity of MPPs and their respective hydrolysates against DPP-IV was assayed using the spectrophotometric method [86]. Samples were subjected to ultrafiltration (centrifugation at 14,000× *g* for 30 min, Laboratory Microcentrifuge Z233 M-2, Hermle USA Inc., Franklin, WI, USA) using centrifugal filters (Amicon^®^ Ultra Centrifugal Filters, Merck, Millipore, Darmstadt, Germany) with a molecular weight cut-off of 3 kDa. Briefly, 0.025 mL of 12 mM glycyl-prolyl-p-nitroanilide substrate was added to 0.025 mL of the sample (i.e., MMP and its respective digest) at the appropriate concentration, dissolved in 100 mM tris-HCl buffer (pH 8.0), in a 96-well microplate. Firstly, the mixture was stirred and incubated at 37 °C for 10 min. Then, the reaction was initiated by adding 0.050 mL of a DPP-IV solution (0.4 mU) and continued at 37 °C for 60 min. Afterwards, 0.1 mL of a 1.0 M acetate buffer (pH = 4.0) was added to the mixture to terminate the reaction. Finally, the absorbance of the sample was measured at 405 nm (iMark Microplate Absorbance Reader; Bio-Rad, Hercules, CA, USA). The percentage of DPP-IV inhibition was calculated using Equation (2).

% _DPP-IV inhibition_ = [(A_S_ − A_SE_)/(A_C_ − A_CE_)] × 100 (2)

where A_S_—absorbance of the sample (MPP or its respective digest); A_SE_—absorbance of the sample with the substrate (enzyme was substituted with buffer); A_C_—absorbance of the mixture containing DPP-IV and the substrate (sample was substituted with buffer); and A_CE_—absorbance of the substrate (sample and DPP-IV were substituted with buffer) [87]. The assay was carried out in triplicate for the 5 concentrations of the samples, ranging between 0.1 and 10 mg/mL.

(3) DPPH^•^ (i.e., 2,2-diphenyl-β-picrylhydrazyl) radical scavenging activity

The DPPH^•^ radical scavenging activity of MPPs and/or their respective hydrolysates was assayed according to Wu et al. [88] with slight modifications. Briefly, MPPs or their hydrolysate solutions (0.5 mL) were mixed with the same volume of DPPH (0.15 mM) in 95% ethanol. Then, the absorbance of the mixture was measured (λ = 517 nm; GENESYS 150, Thermo Scientific, Waltham, MA, USA). The assay was performed in triplicate for the 5 concentrations of the sample, ranging from 0.75 to 3.0 mg/mL. The percentage of DPPH^•^ radical scavenging activity was calculated using Equation (3):

% _DPPH• radical scavenging activity_ = [(A_control_ − A_sample_)/A_control_] × 100% (3)

where A_control_—absorbance of the mixture containing water (instead of the sample) and a DPPH solution, and A_sample_—absorbance of the mixture containing the sample and a DPPH solution.

(4) ABTS^•+^ (i.e., 2′-azino-bis(3-ethylbenzothiazoline-6-sulfonic acid) diammonium salt) radical scavenging effect

ABTS^•+^ radical scavenging activity of MPPs and/or their respective hydrolysate was measured using the method described by Re et al. [89] with modifications proposed by Borawska et al. [90]. Firstly, the cation radical solution containing 7.0 mM ABTS^•+^ and 2.45 mM potassium persulfate (K_2_S_2_O_8_) was prepared by overnight incubation (16.0 h) of the mixture. Then, the solution was diluted using a 10.0 mM phosphate-buffer saline solution (PBS; pH = 7.4) to achieve the absorbance value of 0.700 ± 0.020 (λ = 734 nm). Afterwards, 10.0 µL of the sample and 1.0 mL of a diluted ABTS^•+^ solution preheated to 30 °C were mixed together for 30 s, and then incubated in the dark for 6 min. Finally, the absorbance was measured at 734 nm (GENESYS 150, Thermo Scientific, Waltham, MA, USA). The assay was performed in triplicate for the 5 concentrations of the sample, ranging from 1.0 to 10.0 mg/mL. The percentage of ABTS^•+^ radical scavenging activity was calculated using Equation (4) [91]:

% _ABTS•+ radical scavenging activity_ = [(A_control_ − As_ample_)/A_control_] × 100% (4)

where A_control_—absorbance of an ABTS^•+^ solution without the sample (buffer instead), and A_sample_—absorbance of the sample.

(5) Ferric Reducing Antioxidant Power (FRAP)

The Benzie and Strain method [92] was applied to measure the FRAP effect of milk preparations and their hydrolysates. A FRAP reagent (1.425 mL) was first preincubated at 37 °C for 30 min, and then 0.075 mL of the water-soluble sample solution was added. The whole mixture was vigorously stirred and incubated in the dark (37 °C; 30 min). Then, it was centrifuged (10,000× *g*; 3 min; 21 °C) and subjected to absorbance measurements (λ = 593 nm; GENESYS 150, Thermo Scientific, Waltham, MA, USA). Analyses were carried out in triplicate for five concentrations of the sample, ranging from 10.0 to 25.0 mg/mL each. The rate of ferric reducing power was calculated using Equation No. 5 adapted from Venskutonis et al. [93]:

_% FRAP_ = [(A_sample_ − A_blank_)/A_sample_] × 100% (5)

where A_sample_—absorbance of the sample (i.e., FRAP reagent with MPP), and A_blank_—absorbance of the blank (i.e., FRAP reagent and water).

(6) IC50 values for specific bioactivity of MPPs and their hydrolysates

IC_50_ values tantamount to the sample concentrations (mg/mL) corresponding to their half-maximal activity (i.e., bioactivities described above) were quantified using GraphPad Prism^®^ v. 5.02 for Windows (GraphPad Software, Boston, MA, USA) [94]. The computations were carried out using the program options: “Nonlinear regression” → “Dose–response curves—Inhibition” → “inhibition (log) vs. normalized response—variable slope”, and the values obtained included a standard error (at a 95% confidence interval). According to the instructions for IC_50_ estimation, at least five different concentrations of MPPs and/or their respective hydrolysates were applied [19,20].

(7) Identification of Peptides in MPPs and Their Hydrolysates

A total of 1 mg of a lyophilized sample was dissolved in 1000 µL of solution A (water with 0.1% FA). Then, the samples were centrifuged (Hermle Z 233, M-2, HERMLE LaborTechnik GmbH, Wehingen, Germany) at 10,000 rpm for 10 min [46] and afterwards they were moved to Chromacol test tubes. The injection volume was 20 µL.

Peptide identification analyses were carried out according to Darewicz et al. [33] with slight modifications, using an LCMS-9030 device (Shimadzu^®^, Tokyo, Japan) with quadrupole time-of-flight (Q-ToF) and electrospray ionization (ESI). It comprises two LC-30AD pumps, a DGU-20A5R degasser, an SIL30ACMP autosampler, a CTO-20AC thermostat, a CBM-20A controller, an NGM-11 nitrogen generator, and an SF2FF compressor. A bioZen XB-C18 column with a SecurityGuard ULTRA Cartridges pre-column (both from Phenomenex^®^, Torrance, CA, USA) was used for the separation of the samples. The column parameters were as follows: dimension—100 × 2.1 mm, resin diameter—1.6 µm, and pore diameter—100 Å. Column temperature was 40 °C. Separations were carried out in acetonitrile (ACN) gradient. Solvent A contained 0.1% [*v*/*v*] of formic acid (FA) in water, whereas solvent B contained 0.1% of FA in ACN. The total flow rate was 0.2 mL/min, including 5.0% of solvent B as the starting concentration. Concentration gradients of solvents A and B applied for peptide identification are provided in Table 8.

Data were analyzed using the LabSolutions program v. 5 (Shimadzu, Tokyo, Japan). The following ESI parameters were used: the flow rate of nebulizing gas—3.0 L/min, the flow rate of heating gas—10.0 L/min, interface temperature—300 °C, and temperature of eluent evaporation—526 °C. Ion Guide parameters were as follows: the flow rate of drying gas—10.0 L/min, heat block temperature—400 °C, and desolvation line temperature—250 °C. The voltage of the sub-interface was 4.00 kV.

Mass spectrometry and tandem mass spectrometry methods (MS and MS/MS, respectively) were applied to identify peptides in the samples. The analyses were carried out in the 500–2000 *m*/*z* range between 0 and 60th minute. The input precursor mass during MS^2^ (MS/MS) analysis corresponded to the searched mass of the peptide (M + H^+^). Collision Energy was 20 ± 10 eV, maximal loop time was 1.0 s, which was 0.1 s per analysis. Peptide identification in the sample consisted of the comparison of MS/MS spectra with those found in the METLIN database [65] and searching for distinctive fragmentation ions.

## 4. Conclusions

Based on an in silico analysis, 59 peptides were produced from bovine milk protein sequences upon the combined action of pepsin, trypsin, and chymotrypsin. The great majority of them were ACE and DPP-IV inhibitory peptides, whereas only five peptides exerted the antioxidative effect. The applied enzymes were more effective in releasing DPP-IV inhibitors than ACE inhibitory peptides.

MCC was the most active against ACE (IC_50_ = 1.856 mg/mL), whereas MBP D showed the lowest ACE-inhibiting activity (IC_50_ = 7.627 mg/mL). MCC was also the most active preparation among the non-digested MPPs. The non-digested SPC showed the lowest activity (IC_50_ = 3.738 mg/mL). All MPPs, except MCC, inhibited DPP-IV. Among MPPs not subjected to the simulated digestion, MBP was the most active against DPP-IV (IC_50_ = 0.561 mg/mL). MBP D exerted the strongest DPP-IV inhibitory effect among all samples (before and after digestion; IC_50_ = 0.0067 mg/mL). Regardless of the test applied, all MPPs possessed antioxidative activity. MBP D possessed the highest ABTS^•+^ radical scavenging activity (IC_50_ = 2.754 mg/mL), and SPC D elicited the strongest DPPH^•^ radical scavenging effect (IC_50_ = 1.238 mg/mL).

ACE and DPP-IV inhibitory peptides were identified in all MPPs. Thirteen ACE inhibitors had known IC_50_ values, and four of them (IW, PR, ST, VY) had IC_50_ values below 10 µM. IW and PR were identified in SPC after simulated duodenal digestion, whereas ST and VY were found in all digested MPPs. Ten DPP-IV identified peptides were characterized with known IC_50_ values. Four sequences were the strongest DPP-IV inhibitors, namely: VL, IPA, IPM, and VPL, with IC_50_ ranging from 15.80 to 74.00 µM. VL and IPM were identified in all duodenally digested samples, whereas IPA and PPL were identified in the casein-containing digested MPPs.

Although RP-HPLC-UV showed that casein was less susceptible to hydrolysis than whey proteins, more bioactive peptides were identified in the casein-containing MPPs.

Based on the results achieved for all MPPs, MBP D can especially be considered a functional agent supporting health-beneficial functions of dairy products.

## Figures and Tables

**Table 1:** Values of parameters describing the effectiveness of proteolysis of bovine milk proteins using pepsin, trypsin, and chymotrypsin in producing ACE and DPP-IV inhibitors.

Protein	A_E_ *		W		B_E_		V	
	Inhibitory Activity Against							
	ACE	DPP-IV	ACE	DPP-IV	ACE	DPP-IV	ACE	DPP-IV
α_s1_-CN, B	0.0452	0.0804	0.0825	0.1311	0.0018	0.0004	0.0765	0.8077
α_s2_-CN, A	0.0676	0.0991	0.1471	0.1419	0.0043	0.0001	0.1587	0.0298
β-CN, A1	0.0526	0.1675	0.082	0.2084	0.0012	0.0002	0.0263	0.1385
κ-CN, A	0.1105	0.1579	0.2165	0.2027	0.0047	0.0002	0.0996	0.1429
α-La, A	0.0775	0.0915	0.1865	0.1368	0.0049	0	0.2235	0
β-Lg, A	0.0678	0.1186	0.1186	0.181	0.0018	0.0003	0.0195	0.3353
BSA	0.0428	0.0939	0.1125	0.1588	0.0029	0.0001	0.1373	0.5195
Lf	0.0452	0.0876	0.1026	0.1423	0.0058	0.0002	0.2706	0.4435

* Abbreviations: A_E_—the frequency of release of fragments with a given activity by selected enzymes; W—the relative frequency of release of fragments with a given activity by selected enzymes; B_E_—activity of fragments potentially released by proteolytic enzyme; V—relative activity of fragments potentially released by proteolytic enzyme (enzymes); ACE—angiotensin converting enzyme; DPP-IV—dipeptidylpeptidase IV; α_s1_-CN, B—α_s1_-casein, genetic variant B; α_s2_-CN, A—α_s2_-casein, genetic variant A; β-CN, A1—β-casein, genetic variant A1; κ-CN, A—κ-casein, genetic variant A; α-La, A—α-lactalbumin, genetic variant A; β-Lg, A—β-lactoglobulin, genetic variant A; BSA—bovine serum albumin; Lf—lactoferrin.

**Table 2:** The average protein content [%] of MPPs and their respective hydrolysates.

Average Percentage of Protein ^1^ in:					
MCC ^2^		MBP ^3^		SPC ^4^	
0 ^5^ ± SD ^6^	D ^7^ ± SD	0 ± SD	D ± SD	0 ± SD	D ± SD
68.67 ± 4.50	32.33 ± 1.89	56.67 ± 1.70	26.33 ± 0.94	53.67 ± 0.94	27.33 ± 0.47

^1^ The assay was made for 1 mg/mL sample; percentage content of protein was read from the standard curve as the mean value of three measurements; ^2^ MCC—micellar casein concentrate; ^3^ MBP—MCC with ultrafiltrated buttermilk permeate; ^4^ SPC—serum protein concentrate; ^5^ 0—sample before simulated digestion phase; ^6^ SD—standard deviation; ^7^ D—sample after duodenal digestion phase.

**Table 3:** Area percentages under peaks in the chromatograms assigned to selected time segments.

Sample	Percentage of Areas Under Peaks in the Time Segments [%]			
Retention Times [min]	0.00–9.99	10.00–19.99	20.00–54.99	55.00–70.00
MCC *	95.421	1.110	1.107	2.363
MCC D	95.977	2.505	1.175	0.343
MBP	96.871	1.305	0.860	0.964
MBP D	95.538	2.821	1.341	0.300
SPC	92.570	1.131	0.505	5.794
SPC D	95.576	3.007	1.082	0.335
SM	91.064	0.000	5.121	3.815
SM D	91.253	0.029	7.941	0.777

* Abbreviations: MCC—micellar casein concentrate before simulated digestion; MCC D—MCC subjected to the duodenal phase of simulated digestion; MBP—MCC with buttermilk permeate ultrafiltrated before simulated digestion; MBP D—MBP subjected to the duodenal phase of simulated digestion; SPC—serum protein concentrate before simulated digestion; SPC D—SPC subjected to the duodenal phase of simulated digestion; SM—skim milk; SM D—SM subjected to the duodenal phase of simulated digestion; peak area until the 70th min was defined as 100%.

**Table 4:** Bioactivities of MPPs and their respective hydrolysates.

Sample	IC_50_ [mg/mL] *				
	Inhibition of		Antioxidative Power Towards		
	ACE	DPP-IV	ABTS^•+^	DPPH^•^	FRAP
MCC	1.856	n.d.	3.444	1.645	16.880
MCC D	2.897	0.404	4.673	1.481	14.990
MBP	2.307	0.561	4.144	1.342	31.120
MBP D	7.627	0.0067	2.754	1.687	20.140
SPC	3.738	1.693	6.244	1.246	14.470
SPC D	4.143	0.512	4.616	1.238	13.720

* Abbreviations: IC_50_—the concentration of the sample corresponding to its half-maximal bioactivity (mg/mL); ACE—angiotensin converting enzyme; DPP-IV—dipeptydylpeptidase IV; ABTS^•+^—2,2′-azino-bis(3-ethylbenzotialozline-6-sulfonic acid) radical cation; DPPH^•^—2,2-diphenyl-*β*-picrylhydrazyl radical; FRAP—Ferric Reducing Antioxidant Power; MCC—micellar casein concentrate before simulated digestion; MCC D—MCC subjected to the duodenal phase of simulated digestion; MBP—MCC with buttermilk permeate ultrafiltrated before simulated digestion; MBP D—MBP subjected to the duodenal phase of simulated digestion; SPC—serum protein concentrate before simulated digestion; SPC D—SPC subjected to the duodenal phase of simulated digestion.

**Table 5:** Bioactive peptides identified in MPPs before and after duodenal simulated digestion, including their retention times, *m*/*z*, and protein source.

*m*/*z* (M + H)+	Sequence ^1^	Retention Time [min] of Peptide Identified in:						Protein Source ^7^
		MCC ^2^		MBP ^3^		SPC ^4^		
		0 ^5^	D ^6^	0	D	0	D	
163.0714	SG	-	-	1.28	-	1.25	-	α_s1_-CN; κ-CN; α-La
175.1078	VG	-	1.27	-	1.27	-	1.27	α-La; Lf; BSA
187.1078	PA	-	1.45	-	1.45	-	1.45	κ-CN; β-Lg
189.1234	IG	-	2.19	-	2.19	-	2.19	α_s1_-CN; α_s2_-CN
189.1234	VA	4.52	4.52	4.52	4.52	-	4.52	α_s1_-CN; α_s2_-CN; β-CN; κ-CN; β-Lg; Lf; BSA
205.1183	VS	4.58	4.53	4.63	4.62	-	4.62	BSA
207.0976	ST	-	2.83	-	2.87	-	2.87	α_s1_-CN; α_s2_-CN; κ-CN; α-La; Lf; BSA
215.1391	VP	-	-	2.05	1.9	-	-	α_s2_-CN; Lf
217.1183	PT	-	-	-	-	-	1.52	α_s2_-CN; β-CN; κ-CN; β-Lg; Lf; BSA
219.1340	SL	-	2.04	-	2.03	-	2.01	β-CN; β-Lg; Lf; BSA
219.1340	VT	-	1.17	-	1.12	-	-	κ-CN; β-Lg; Lf; BSA
229.1547	PL	-	3.89	-	3.82	-	3.77	α_s1_-CN; β-CN; β-Lg; Lf; BSA
229.1547	IP	-	3.89	-	3.82	-	3.77	κ-CN
230.1136	PN	-	1.31	-	1.31	-	1.31	Lf; BSA
231.1704	VL	-	2.68	-	2.61	-	-	α_s1_-CN; α_s2_-CN; β-CN; κ-CN; β-Lg; Lf; BSA
234.1449	SK	-	1.89	-	1.90	-	-	α_s1_-CN; α_s2_-CN; β-CN; Lf
244.1656	PK	-	1.32	-	1.32	-	1.27	α_s1_-CN; α_s2_-CN; β-CN; Lf; BSA
246.1449	IN	-	-	-	-	-	2.49	β-CN; κ-CN; α-La
247.1111	PM	-	2.83	-	2.83	-	2.86	α_s1_-CN; β-Lg
249.1268	VM	-	1.55	-	1.58	-	1.57	β-CN; BSA
253.1296	PH	-	1.20	-	1.20	-	1.21	β-CN; κ-CN; α-La; BSA
261.1445	IE	-	2.37	-	2.36	-	2.36	β-CN; κ-CN; BSA
263.1391	PF	-	6.96	-	6.94	-	6.94	α_s1_-CN; β-CN; κ-CN
269.1609	IH	-	2.22	-	2.22	-	2.28	α_s1_-CN; β-CN; κ-CN
272.1718	PR	-	-	-	-	-	1.20	Lf
274.1874	VR	-	3.59	-	-	-	-	α_s2_-CN; β-CN; κ-CN; β-Lg; Lf; BSA
279.1340	PY	-	3.07	-	3.15	-	3.17	κ-CN; Lf; BSA
281.1496	VY	-	4.52	-	4.50	-	4.53	α_s2_-CN; β-CN; β-Lg
288.2031	IR	-	5.94	-	5.96	-	-	κ-CN; β-Lg; Lf
292.1292	SW	-	1.28	-	1.31	-	1.28	β-CN; Lf
300.1918	IPA	-	3.45	-	3.45	-	-	β-Lg
302.1500	PW	-	5.82	-	5.82	-	5.82	α_s2_-CN
318.1813	IW	-	-	-	-	-	2.32	α_s2_-CN; α-La; Lf
326.2075	PPL	-	15.01	-	14.95	-	-	β-CN; BSA
328.2231	VPL	-	7.71	-	7.76	-	7.59	α_s1_-CN
360.1952	IPM	-	11.39	-	10.45	-	10.48	Lf
Number of peptides	36	2	30	4	30	1	28	

^1^ In the case of Leu- and Ile-containing peptides, there is a possibility of the occurrence of the isomers that characterize the same mass of fragmentation ions; ^2^ MCC—micellar casein concentrate; ^3^ MBP—MCC with buttermilk permeate ultrafiltrated; ^4^ SPC—serum protein concentrate; ^5^ 0—sample before simulated digestion, ^6^ D—sample subjected to duodenal simulated digestion; ^7^ protein source: α_s1_-CN—α_s1_-casein (UniProt ID: P02662); α_s2_-CN—α_s2_-casein (UniProt ID: P02663); β-CN—β-casein (UniProt ID: P02666); κ-CN—κ-casein (UniProt ID: P02668); α-La—α-lactalbumin (UniProt ID: P00711); β-Lg—β-lactoglobulin (UniProt ID: P02754); Lf—lactoferrin (UniProt ID: P24627); BSA—bovine serum albumin (UniProt ID: P02769).

**Table 6:** The number of peptides showing specific bioactivity identified in duodenally digested bovine milk protein preparations.

Activity	MCC D *	MBP D	SPC D
Total	30	30	28
Angiotensin I-converting enzyme (ACE) (EC 3.4.15.1) inhibition	10	11	11
Dipeptidyl peptidase IV (DPP-IV) (EC 3.4.14.5) inhibition	27	27	23
Antioxidative	3	3	2

* Abbreviations: MCC D—MCC subjected to the duodenal phase of simulated digestion; MBP D—MBP subjected to the duodenal phase of simulated digestion; SPC D—SPC subjected to the duodenal phase of simulated digestion.

**Table 7:** The gradients of solvents A and B used in RP-HPLC-UV/VIS analysis of the samples.

Stage	Time [min]	Solvent [%]	
		A	B
Separation	0	100	0
	60	60	40
Washing	65	0	100
	70	0	100
Column equlibration	71	100	0
	80	100	0

**Table 8:** Concentration gradient of solvents A and B applied for peptide identification in MPPs and their respective hydrolysates.

Stage	Time [min]	Solvent [%]	
		A	B
Separation	0:00	95	5
	40:00	65	35
Column washing	40:01	65	35
	41:00	0	100
	46:00	0	100
Column equilibration	46:01	0	100
	47:00	95	5
	60:00	95	5

## Data Availability

The original contributions presented in this study are included in the article/Supplementary Material. Further inquiries can be directed to the corresponding author.

## Supplementary Materials

The following supporting information can be downloaded at https://www.mdpi.com/article/10.3390/ijms26094323/s1.

## Abbreviations

The following abbreviations are used in this manuscript:

α_s1_-CN, B	α_s1_-casein, genetic variant B
α_s2_-CN, A	α_s2_-casein, genetic variant A
β-CN, A1	β-casein, genetic variant A1
κ-CN, A	κ-casein, genetic variant A
α-La, A	α-lactalbumin, genetic variant A
β-Lg, A	β-lactoglobulin, genetic variant A
ABTS	2,2′-azino-bis(3-ethylbenzotialozline-6-sulfonic acid
ACE	Angiotensin converting enzyme (EC 3.4.15.1)
ACN	Acetonitrile
BSA	Bovine serum albumin
IC_50_	Tantamount to the sample concentrations (mg/mL) corresponding to their half-maximal activity
BIOPEP-UWM	BIOPEP-UWM database of protein and bioactive peptide sequences
DPP-IV	Dipeptidylpeptidase-IV (EC 3.4.14.5)
DPPH	2,2-diphenyl-*β*-picrylhydrazyl
FA	Fluoroacetic acid
FRAP	Ferric Reducing Antioxidant Power
Lf	Lactoferrin
MBP	MCC with ultrafiltrated buttermilk permeate
MBP D	MBP subjected to the duodenal phase of simulated digestion
MCC	Micellar casein concentrate
MCC D	MCC subjected to the duodenal phase of simulated digestion
SPC	Serum protein concentrate
SPC D	SPC subjected to the duodenal phase of simulated digestion
MPP	Milk Protein Preparation
MS	Mass spectrometry
MS/MS	Tandem mass spectrometry
PEPT1	Peptide Transporter 1
PEPT2	Peptide Transporter 2
RP-HPLC UV/Vis	Reversed-phase high-performance liquid chromatography with UV-visible Spectroscopy
SM	skim milk
SM D	SM subjected to the duodenal phase of simulated digestion
