# Supplementary material for: Bovine Milk Protein-Derived Preparations and Their Hydrolysates as Sources of ACE-Inhibitory, DPP-IV-Inhibitory, and Antioxidative Peptides Analyzed Using in Silico and in Vitro Protocols
Source: Int J Mol Sci. 2025 May 1;26(9):4323. doi: 10.3390/ijms26094323 (PMC12073076; doi:10.3390/ijms26094323)
Supplement: Supplementary file 1 [file ijms-26-04323-s001.zip › ijms-3548695-supplementary.pdf]

SUPPLEMENT

Bovine milk protein-derived preparations and their hydrolysates as sources of ACE-inhibitory, DPP-IV-inhibitory and antioxidative peptides analyzed using in silico and in vitro protocols

Anna Iwaniak [1,*], Piotr Minkiewicz [1], Damir Mogut [1], Justyna Borawska-Dziadkiewicz [1], Justyna Żulewska [2] and Małgorzata Darewicz [1]

[1] Department of Food Biochemistry, Faculty of Food Science, University of Warmia and Mazury in Olsztyn, Pl. Cieszyński 1, 10-719 Olsztyn-Kortowo, Poland; ami@uwm.edu.pl (A. I.); minkiew@uwm.edu.pl (P.M.); damir.mogut@uwm.edu.pl (D.M.); justyna.borawska@uwm.edu.pl (J.B.-D.); darewicz@uwm.edu.pl (M.D.)
[2] Department of Dairy Science and Quality Management, Faculty of Food Science, University of Warmia and Mazury in Olsztyn, Oczapowskiego 7, 10-719 Olsztyn-Kortowo, Poland; justyna.zulewska@uwm.edu.pl
* Correspondence: ami@uwm.edu.pl

Table S1. List of peptides potentially released from bovine milk proteins upon the simulated coupled action of pepsin, trypsin and chymotrypsin. Each peptide is assigned to its ID number in the BIOPEP-UWM database [**35, 36**], including links to peptide data in this database.

**Sequence**[1]	**Function**				
	**ACE Inhibitor**[2]	**IC$_{50}$ [µM]**[3]	**DPP IV-inhibitor**[2]	**IC$_{50}$ [µM]**[3]	**Antioxidative**[2]
CF	7751	-	-	-	-
IA	7562	-	8525	-	-
***IE**	7827	-	-	-	-
IF	7593	-	-	-	-
***IG**	7595	**1200.00**	-	-	-
***IH**	-	-	8800	-	-
IL	9079	-	8802	-	-
IM	-	-	8803	-	-
***IN**	-	-	8804	-	-
***IP**	7581	**130.00**	8501	**410.00**	-
IQ	-	-	8805	-	-

***IR**	3258	**695.00**	8806	-	8215
***IW**	7544	**4.70**	8807	-	-
IY	3383	-	-	-	7873
***PA**	-	-	3179	-	-
***PF**	-	-	8854	-	-
PG	7625	-	8855	-	-
***PH**	7843	-	8856	-	-
***PK**	-	-	8858	-	-
***PL**	7513	**337.42**	8638	-	-
***PM**	-	-	8859	-	-
***PN**	-	-	8860	-	-
PQ	7837	-	8861	-	-
***PR**	3537	**4.10**		-	-
PS	-	-	8862	-	-
***PT**	-	-	8863	-	
***PW**	-	-	8865	-	8190
***PY**	-	-	8866	-	-
SF	7865	-	8891	-	-

***SG**	7618	**8500.00**	-	-	-
SH	-	-	8892	-	-
***SK**	-	-	8894	-	-
***SL**	-	-	8560	**2517.08**	-
***ST**	9184	**4.03**	-	-	-
***SW**	-	-	8896	-	-
***VA**	-	-	3172	**168.24**	-
VD	-	-	8915	-	-
VE	7829	-	8916	-	-
VF	3384	-	8917	-	-
***VG**	7594	**1100.00**	8918	-	-
VH	-	-	8919	-	-
VK	7558	-	8921	-	-
***VL**	-	-	8922	**74.00**	-
***VM**	-	-	8923	-	-
VN	-	-	8924	-	-
***VP**	7587	**420.00**	3181	**880.00**	-
VQ	-	-	8925	-	-

***VR**	7628	**52.80**	8594	**826.10**	-
***VS**	-	-	8926	-	-
***VT**	-	-	8927	-	-
VW	3486	-	8928	-	8461
***VY**	3492	**7.10**	8929	-	8224
***IPA**	3507	**141.00**	8304	**49.00**	-
***IPM**	-	-	9233	**69.50**	-
IPY	7803	-	-	-	-
***PPL**	-	-	8652	**390.14**	-
PSY	7559	-	-	-	-
VPK	3975	-	-	-	-
***VPL**	-	-	8347	**15.80**	-

[1] - bold and asterisk – peptide identified in the MPPs and/or their appropriate hydrolysates (see Results and Discussion), [2] – BIOPEP-UWM ID of peptide, corresponding to the given function; [3] –IC_{50} [μM] (if any) of the identified peptide based on literature data taken from the BIOPEP-UWM database.

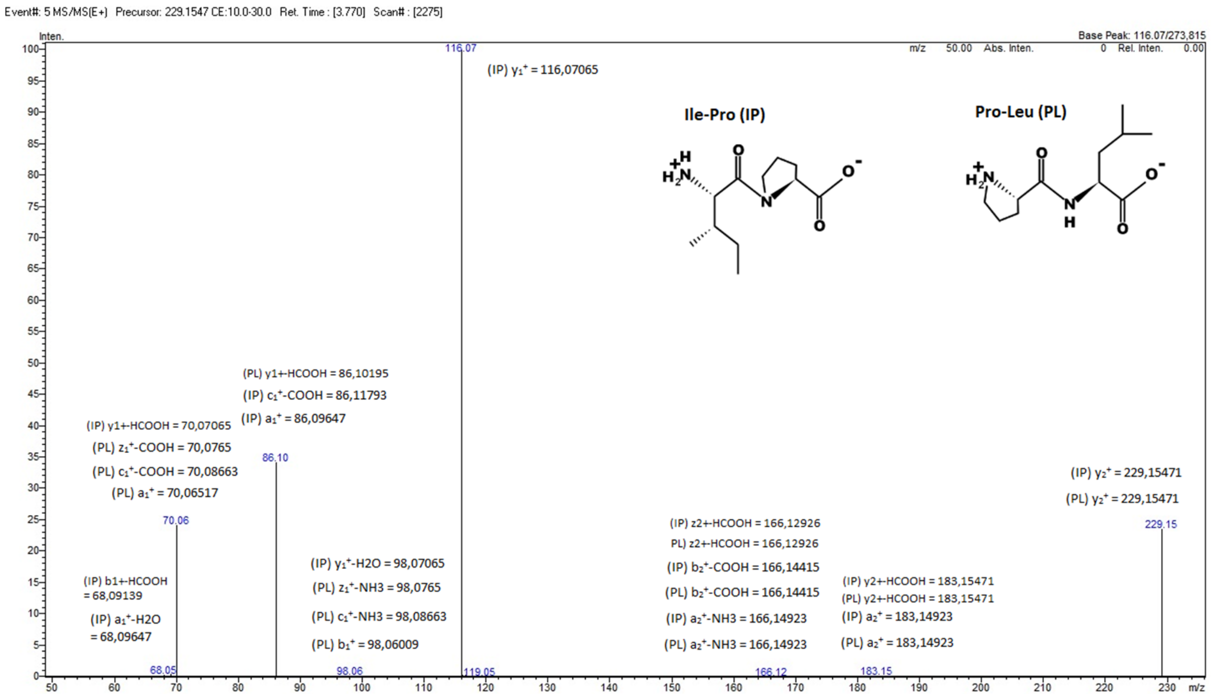

Figure S1. MS/MS spectrum of PL and IP peptide. Fragmentation ions were systemized according to the nomenclature introduced by Roepstorff and Fohlman [**60**]. Asterisks denote the fragmentation ions present in reference spectra of the same peptide found in the METLIN database [**65, 66**].
